# Supplementary material for: Are carbon emissions trading and green financial instruments synergistic? -Comprehensive quantitative research based on content analysis
Source: PLoS One. 2024 Mar 7;19(3):e0298601. doi: 10.1371/journal.pone.0298601 (PMC10919601; doi:10.1371/journal.pone.0298601)
Supplement: S1 Data — (ZIP) [file pone.0298601.s001.zip › Data/地级市Z_F工作报告.zip!/░▓╗╒/╨√│╟╩╨/└┤╫╘╣1⁄2╓┌║┼í╛┬φ┐╦╩2╛▌═°í┐_2009.docx]

　第二届人民代表大会第六次会议上

　　宣城市人民政府市长 虞爱华

各位代表：

　　现在，我代表市人民政府，向大会作政府工作报告，请予审议，并请市政协委员和其他列席人员提出意见。

　　一、2009年工作回顾

　　刚刚过去的一年，在省委、省政府和市委的坚强领导下，全市人民深入贯彻落实科学发展观，积极应对国际金融危机，抢抓国家扩大内需政策机遇，紧紧围绕保增长、保民生、保稳定大局，迎难而上、主动作为，远谋近施、自我加压，圆满完成了年度目标任务，经济社会延续了近年来良好的发展势头，在跨越发展进程中迈出更加坚实的步伐。

　　初步统计，全年实现生产总值432.8亿元，同比增长13.6%，增幅居全省第6位，在全省的位次为近年来最好。财政总收入60.4亿元，增长25.9%，净增12亿元，增量为历史最大，增幅居全省第5位；其中地方财政收入35.4亿元，增长28.2%，总量和增幅均居全省第7位。固定资产投资600.8亿元，总量在2008年跃过300、400亿的基础上，跃过500、600亿，居全省第4位，增长44.1%，增幅居全省第5位。社会消费品零售总额167.1亿元，增长18.8%。城镇居民人均可支配收入13302元，增长12.6%，增幅居全省第1位。预计农民人均纯收入5700元，增长12%，高于全省1200元左右。万元生产总值能耗下降5.5%，化学需氧量、二氧化硫排放量分别减少3.4%和4.7%。受金融危机影响，对外贸易有所下降，完成外贸进出口总额4.66亿美元，降幅比全省低5.6个百分点。基本实现了主要指标增幅进入全省第一方阵的目标。

　　(一)积极应对金融危机，着力保持经济平稳较快增长。一是坚持不懈抓投入。始终坚持谋划项目、积极争取投资，搭建平台、努力扩大融资，优化环境、大力招商引资。全年累计争取中央扩大内需投资7.2亿元，与央企签订合作项目11个，列入省“861”行动计划项目139个。做大做实了市国投公司，注册资本金20亿元，总资产近60亿元，全年累计融资16.8亿元。先后与4家省金融机构签订银政战略合作框架协议，累计取得意向性信用额度350亿元。中鼎股份增发成功，融资近2.5亿元。坚持“走出去”、“请进来”，先后在杭州、台湾等地举办招商推介会15次，充分利用中博会等活动开展招商引资，取得明显成效。全年实际到位省外资金345亿元，增长59%，总量和增幅均居全省第4位；实际到位外资1.54亿美元，增长19.6%，增幅居全省第6位。二是多措并举抓帮扶。出台了促进工业经济平稳较快发展“新八条”，设立了“中小企业发展专项扶持资金”和出口企业孵化基金。组建了市金融办。积极开展银企对接，签订授信协议67亿元，当年投放55亿元。组织开展了7场中小企业金融服务日和“小企业金融超市”活动，累计拉动全市签约和发放中小企业贷款37.6亿元。加强中小企业担保机构建设，在保金额超过20亿元，是上年的3倍。批准筹建小额贷款公司15家，其中开业10家，批建数量居全省第2位，累计发放贷款6亿元。全年新增贷款80.6亿元，是去年的2.9倍，增长40.4%，居全省第2位。各类保险机构实现保费15亿元。建立了市领导联系帮扶企业、市直部门定点联系企业、百名干部进驻企业帮扶等制度，解决了企业发展中的一系列困难与问题。支持中鼎、宣酒、司尔特、和威等企业做大做强。认真落实社保“五缓四降三补贴”政策，累计减免退抵税款以及取消和降低行政事业性收费8.6亿元。三是统筹兼顾抓调度。坚持对市本级抓具体、抓项目，对县市区抓调度、抓氛围，实行月调度、季分析、半年点评制度。每个月发展较慢的县(市、区)政府主要负责人列席市政府常务会议，共同分析原因，研究对策。实行市政府工作部门分口汇报工作制度，加强对重点行业、重点企业的运行监测分析，强化对市直单位招商引资的调度和考核，及时、全面地掌握经济运行情况，有针对性地提出解决办法。

　　(二)强力推进开发区建设。市本级和各县(市、区)通过抓开发区建设，大力推进工业化进程。全市工业化率由34.2%提高到34.5%。新增规模企业169户，规模企业总数达到1081户，实现规模工业增加值153.1亿元，增长27.2%，增幅居全省第4位，规模工业实现利润增长82%，增幅位居全省第4。完成工业固定资产投资276.6亿元，增长50%，总量居全省第4位，增幅居全省第2位。市开发区的路、水、电等基础设施建设进一步加快，投资服务功能进一步强化。13个市直部门向驻开发区分局授权。项目建设加快，48个项目相继开工，其中9个项目建成投产；新签工业项目31个，其中亿元以上项目16个。“飞地经济”开始起步，与浙江玉环22家企业签订了玉环工业园框架性协议，有3家企业开工建设。全年实现工业增加值7.9亿元，增长23.1%；财政收入2.5亿元，增长33.9%。各县(市、区)开发区发展也进一步加快。宁国开发区跻身省级电子信息产业专业园区、信息化和工业化融合试验区、自主创新综合改革试验区行列。宣州开发区初步形成“八纵三横”路网，多家企业落户。广德开发区开发面积已达15平方公里。郎溪、泾县、绩溪、旌德开发区建设力度进一步加大。郎溪鸿泰特种钢技改项目、泾县隆鑫精密件铸造一期工程建成投产，司尔特生态化工产业园和博瑞特特种设备产业园等重大项目继续推进，郎溪动力源生产基地、广德通用汽车研发试验中心和金鹏科技、绩溪异型链生产基地、旌德益三信息产业园等重大项目开工建设，宁国医疗器械产业园、绩溪抽水蓄能电站、旌德双和教育项目已进入实质性推进阶段。

　　(三)继续加强新农村建设。全年粮食总产133.7万吨，超额完成110万吨目标任务。农业产业化水平得到提升，农产品加工产值超过200亿元，增长20%。新增年销售收入10亿元和5亿元龙头企业各1家、亿元龙头企业6家。新增省级示范专业合作社20家，占全省的五分之一。提前完成“552强龙工程”和“十会百社工程”目标。“家禽强市”建设稳步推进，养殖规模达到2亿多羽，占全省的近四分之一。水稻生产提升行动继续实施，建立55万亩水稻核心示范区和11个万亩粮食高产创建示范片。竹林丰产增效“636”示范工程稳步推进。新增中国名牌农产品1个，新增农产品“三品”认证20个。获得农业部农产品地理标识认证3个。完成“农业信息化推进年”工程建设，初步建成宣城市特色农产品综合信息网。在全省率先建成乡镇土地流转服务中心(站)，耕地流转面积达到26.4万亩，增长67.3%，高于全省平均水平。扩权强镇改革试点工作稳步开展。村级公益事业建设“一事一议”财政奖补试点工作有序推进。集体林权制度主体改革任务基本完成，配套改革初见成效。超额完成人工造林任务，造林面积位居全省前列。水阳江下游“开卡”工程前期工作基本完成。155座中小型病险水库得到除险加固。农村公共服务设施进一步改善。新增水泥路、沥青路610公里，99%的行政村通上水泥路。90%的自然村通宽带。解决了17.6万农村人口饮水安全问题，农村饮水安全覆盖率增长7.4%。广德、绩溪通过全省新农村建设电气化县验收，新建电气化乡镇10个、村75个。完成了20个乡镇综合文化站和268家农家书屋建设任务。新农村建设新增7个示范镇、70个示范村。抗击“八号台风”取得全面胜利。

　　(四)交通建设实现突破。合福高铁项目已经开工，皖赣铁路扩能和电气化改造以及合杭、黄杭高铁前期工作扎实开展。宣城至宁国、宁国至绩溪高速公路开工建设，绩溪至黄山高速公路年度建设任务超额完成，芜雁高速公路建设进展顺利。宁国至千秋关高速公路已经动迁。溧阳至广德高速公路前期工作加速推进。千秋关隧道建成通车。宣南线、老318线宣城至广德段、蔡家桥至谭家桥段、华龙公路等改建项目完成年度建设任务。芜申运河宣城段开工建设。水阳江航道整治工程前期工作进展顺利。南京禄口机场宣城候机楼和城市货站正式投入使用。我市高速公路前期工作受到省政府表彰奖励，农村公路建管养及“村村通”工程建设等工作在全省处于领先位次。

　　(五)城市建设力度加大。全市城镇化率由41.7%提高到43.2%。市本级全年完成城建投资近80亿元，其中基础设施建设投资24.4亿元，公共服务配套设施建设投资23亿元，房地产投资32.3亿元。编制完成13个专项规划。西部新城分区规划通过评审。水阳江大道东南段等10条市政道路和宣中体育场、市妇儿活动中心、花鼓戏基地及殷村水库主体工程竣工使用。9条市政道路和一批市政公用设施正在建设。环城大道、宛溪河、扬子鳄湖整治和梅溪公园建设工程已经开工。北门旧城改造顺利推进。89条背街后巷得到亮化。禁违拆违和市容市貌专项整治成效显著，违法建设蔓延态势得到有效遏制。县城建设全面加快。郎溪、旌德新城区框架初步拉开。广德县城中心实现南移，行政便民中心投入使用。泾县城市建设力度加大，基础设施不断完善。绩溪县沿街建筑徽派立面改造初见成效。市、县污水处理厂全部建成。

　　(六)服务业有新进展。争创“中国优秀旅游城市”获得成功。开展了“旅游接待设施建设年”活动。新增国家4A级景区3家和省级优秀旅游乡镇6家、最佳旅游乡镇2家。调整了敬亭山风景区管理体制。通过央视和新浪网等媒体进行城市形象和旅游产品的整体营销，在全省率先派送旅游优惠券，拉动了旅游消费市场。全年接待国内游客535.5万人次，旅游总收入38.9亿元，分别增长21.5 %和34.6%。“家电下乡”工作位居全省前列。提前两年完成“新网工程”建设规划目标。“万村千乡市场工程”扎实推进。农发行绩溪支行正式营业。徽商银行设立宁国分支机构获得批准。

　　(七)民生工程建设成效明显。累计投入资金13.2亿元，推进省、市42项民生工程，惠及240多万城乡居民。在综合考评中位列全省第4，获得“全省民生工程组织实施杰出贡献奖”。就业再就业工作进一步加强，新增城镇就业3.5万人，下岗再就业近1.3万人，登记失业率3.37%。加强社会保障体系建设，城镇职工基本养老、医疗、失业、工伤、生育和城镇居民医疗保险超额完成省定目标。企业退休人员养老金按时足额发放。农村低保提标扩面工作全面完成。农村五保供养实现应保尽保。31所农村敬老院改扩建工程圆满完成。新型农村合作医疗制度、城乡困难群众医疗救助制度深入实施。城市公交线路进一步延伸。全市开工建设保障性住房33.1万平方米，竣工16.9万平方米，其中，市本级开工建设23万平方米，竣工13.8万平方米，超额完成年度目标任务。

　　(八)社会建设不断进步。各县(市、区)全部通过国家科技进步目标考核。新增省级工程技术研究中心3家、企业技术中心6家，新批认定高新技术企业16家、高新技术产品52项，获得发明专利20项、省级科学技术奖3项。组织开展了科技特派员行动和“百高联百企、科普进万家”志愿者活动。全面实施中小学教育“四个提升”计划。宁国、广德被评为全省义务教育均衡发展先进县。义务教育阶段教师绩效工资全部兑现。市区学校布点规划实施加快，市特教中心新校区建成使用，宣城中学新校区建设和宣城二中扩建工程前期工作进展顺利。高中阶段毛入学率达85.7%，比上年提高4.1个百分点。中等职业教育得到发展，创建省级示范中等职业学校4所，在全省率先实现“县(市、区)都有合格职教中心”的目标。中小学校舍安全工程积极推进。群众性体育活动蓬勃开展，农民体育健身工程稳步实施。扎实开展第三次全国文物普查工作。“宣纸制作技艺”入选联合国人类非物质文化遗产代表作名录。市档案馆晋升为国家二级档案馆。宣城市历史文化展览馆正式开馆。市图书馆在全省率先实行市级图书馆免费开放。广播电视“村村通”工程和有线电视数字整体转换工作进展顺利。市人民医院被核准为三级综合医院。医药卫生体制改革积极推进。加强了手足口病和甲型H1N1流感防控，没有发生重大疫情。低生育水平持续稳定，生育质量稳步提升，出生人口性别比继续保持正常，人口自然增长率控制在3.5‰以内。荣获“全国人口和计划生育综合改革示范市”称号。“保增长、保红线”和矿产资源整合工作受到国土资源部表彰，第二次土地调查通过国家级验收。农村土地整治积极推进。着力加强生态建设，宁国市顺利通过国家级生态示范区创建验收，新创14个全国环境优美乡镇。成功举办了纪念小平同志黄山谈话30周年暨“中国文房四宝之乡文化旅游节”活动，《欢乐中国行——魅力宣城》大型综艺晚会安全有序，充分展示了宣城良好的对外形象，得到了社会各界的广泛好评。涌现出全国道德模范王直，舍己救人的优秀青年曹军、卢帅被评为安徽十大新闻人物，承办了全国道德模范和身边好人现场交流活动。

　　深入推进“法治宣城”建设，认真开展“法律六进”活动，法律服务和法律援助工作力度加大。建立了依法处置“两闹”、“两堵”等扰乱社会秩序行为的联动机制，深入开展领导干部开门接访、带案下访、下行接访等活动，推进信访积案化解工作，解决了一批群众反映强烈的热点难点问题。实施了市、区公安一体化改革，推进了公安信息化、执法规范化、和谐警民关系“三项建设”，基层警务力量得到加强。大力开展社会治安整治行动，打击“黑恶势力”和“两抢一盗”等专项行动取得成效，社会秩序总体稳定。“质量和安全年”活动受到全国表彰。食品药品安全监管得到加强。组织开展了“安全生产年”活动，安全生产态势平稳。国防教育、国防动员、人民防空和双拥优抚工作进一步加强，驻宣部队和民兵预备役人员在抗灾抢险、支持地方经济建设等方面做出了重要贡献。妇女、未成年人、老年人、残疾人权益得到较好保障。慈善事业进一步发展。统计、物价、民族、宗教、外事侨务、新闻出版、气象、防震减灾等工作取得新成绩，工商、税务、烟草等工作取得新进展。

　　(九)政府自身建设进一步加强。扎实开展了学习实践科学发展观活动，查治“六症”、狠抓整改，领导科学发展的能力得到提高。继续开展了民主考评“百名科长”活动，并向公共服务单位和基层站所延伸，政府部门服务意识进一步强化，行政效能进一步提升。考评活动受到广泛关注，《人民日报》头版作了报道，人民网和中安在线专题宣传。加大了行政审批权改革力度，44个市直单位成立行政审批科，25个单位在市政务服务中心单设窗口，19个单位进入政务服务综合窗口，并设立了4个分中心。市政府机构改革基本完成。深入推进政务公开和电子政务，高度重视网络舆情，开通“网络政民互动”公共服务平台，建立了政府“网络发言人”制度。严格执行工作责任制和行政问责制，加强绩效考核和政务督查，推进了各项工作的落实。坚持依法行政，认真执行市人大及其常委会的决定和决议，定期向市人大及其常委会报告工作，自觉接受其法律监督。坚持重大问题同市政协协商，接受民主监督。认真办理人代会议案、代表建议和政协委员提案。加强与各民主党派、工商联和无党派人士的联系，支持工会、共青团、妇联等人民团体工作。认真落实廉政建设责任制，加强审计监督和行政监察，强化纠风专项治理和“百姓热线”工作，严肃查处了一批违法违纪案件。

　　各位代表，过去的一年，挑战比预料的大，困难比预想的多，但取得的成绩比预期的好。一年来，全市上下实打实地谋发展，心连心地干事业，肩并肩地抓落实，用争先进位的生动实践展示了攻坚克难、志在必得的精神状态，用逆势而上的实际成绩推动了经济和社会的跨越发展。今天的宣城，处处涌动着干事创业的热潮，呈现出生机勃勃的景象。这些成绩的取得，得益于省委、省政府和市委的坚强领导，凝聚着全体人大代表、政协委员的智慧和力量，倾注着全市人民的心血和汗水。在此，我代表市人民政府，向全市广大工人、农民、知识分子、干部，向驻宣解放军指战员、武警官兵和政法干警，向各民主党派、工商联、各人民团体，向关心、支持宣城建设发展的中央和省驻宣单位以及社会各界人士，向在我市创业的投资者、建设者，致以崇高的敬意和衷心的感谢！

　　在充分肯定成绩的同时，我们也清醒地看到，在发展中还存在不少困难和问题。主要是：差距在总量，综合实力仍然不强。制约在交通，区位优势难以充分显现。薄弱在工业，缺少牵动力强的大项目。困难在本级，体量不够大，可用财力不足。滞后在城建，中心城区辐射带动能力不强，服务业设施规模小、档次低。落后在观念，思想需要进一步解放，创新意识需要进一步增强。对此，我们一定高度重视，切实加以解决，绝不辜负全市人民的期望和重托。

　　二、2010年工作目标和主要任务

　　2010年对宣城来说意义特殊——不仅是实施“十一五”规划的最后一年，也是谋划“十二五”发展的关键之年；不仅是本届市政府届满之年，更是宣城撤地建市10周年喜庆之年。我们要紧紧围绕“跨越崛起、富民强市”这一目标，紧紧抓住建设皖江城市带承接产业转移示范区和皖南国际旅游文化示范区这一历史性机遇，进一步增强危机感、紧迫感和责任感，坚决领会好、贯彻好市委的决策部署，更加凝聚好、发挥好全市人民的智慧和力量，整体突破，重点发力，继续保持经济社会又好又快发展的强劲势头，力争做全省科学发展的“先遣队”、跨越崛起的“排头兵”。

　　今年工作的总体要求是：坚持以邓小平理论和“三个代表”重要思想为指导，深入贯彻落实科学发展观，认真贯彻中央和全省经济工作会议精神，牢牢把握发展主题、调整主线，着力扩大有效投入，着力调整经济结构，着力承接产业转移，着力深化各项改革，着力保障改善民生，圆满完成“十一五”规划的各项目标任务，为“十二五”发展奠定更加扎实的基础。

　　综合考虑各种因素，建议今年经济社会发展的主要预期目标是：生产总值增长13%以上；财政收入增长20%以上；固定资产投资增长30%以上；规模工业增加值增长25%左右；社会消费品零售总额增长18%以上；实际利用外资增长20%以上；进出口总额增长11%以上；城镇居民人均可支配收入增长10%以上；农民人均纯收入增长8%以上；城镇登记失业率控制在4.2%以内；人口自然增长率控制在5‰以内；万元生产总值能耗、主要污染物排放总量完成“十一五”规划确定的目标。实现上述目标任务，必须着力抓好以下六个方面工作：

　　第一，坚定不移抓有效投入，加速扩张经济总量。当前和今后相当长的一个时期内，我市必须靠投资拉动发展。一是毫不松懈地谋划争取项目。把握国家投资导向，着力加强先进制造业、现代服务业、战略性新兴产业和重要基础设施等领域的项目谋划，重点围绕自主创新和结构调整、节能环保、“三农”、民生等方面，紧盯央企、紧盯世界500强企业、紧盯高科技企业，加大项目谋划、争取和对接力度。二是持之以恒地推进项目建设。在入突入着着力抓好鑫鸿模具及覆盖件、天康地源热泵、中鼎高性能汽车橡胶密封件和宣酒集团扩建、郎溪特种设备制造、广德通用汽车研发试验项目、宁国绿色节能建材、南华高效电机产业化、黄山恒久异型链、泰科铁塔扩建等50个市重点工业项目建设。认真做好与央企合作项目跟踪落实工作，开工建设绩溪抽水蓄能电站。三是想方设法地拓宽融资渠道。进一步拓展信贷资源，优化信贷结构，扩大有效信贷投入，力争新增贷款高于上年水平。强化政银合作、银企对接，继续开展中小企业金融服务日和“小企业金融超市”活动。努力扩大直接融资，力争企业上市取得更大进展。继续做大做实市国投公司，抓好市场化、规范化运作，增强投融资功能，发挥资产运营功能。完善支持金融业发展的政策措施，不断改善金融生态环境。积极引进金融机构。支持商业银行在重点集镇设立分支机构。大力发展小额贷款公司和中小企业担保公司。发展和规范典当业。四是坚持不懈地推进招商引资。继续组织开展高频率、“地毯式”招商推介活动，加大面向长三角地区的招商力度。确保实际利用外资1.85亿美元，实际到位省外资金450亿元。积极推进“飞地经济”发展，建设玉环机电工业园、瑞安工业园，启动上海淀山湖工业城、浙商资本工业园建设。浓厚市直单位招商氛围。完善县(市、区)招商引资考核制度，继续实行重大招商引资项目“绿色通道”、定期通报调度和奖励等制度。加强招商引资培训，提高招商引资能力。五是千方百计地拓展内需外需。认真落实扩大消费的各项政策，深入推进家电、汽车摩托车下乡以及汽车以旧换新。继续加大保障性住房建设力度，计划开工建设32.5万平方米，竣工28.8万平方米。采取多种措施稳定房价，促进房地产业健康发展。继续抓好“万村千乡市场工程”和“农超对接”项目建设，提高“新网工程”规范化和标准化水平。继续抓好进出口企业孵化工作，加强对骨干出口企业的跟踪服务，努力实现外贸稳中有升。

　　第二，坚定不移抓结构调整，不断提高发展质量。把先进制造业和现代服务业放在更加突出位置来抓，通过引进高新技术产业促进结构调整。一是大力发展先进制造业。认真组织实施装备制造、轻工、纺织、化工、钢铁、有色金属六大产业调整与振兴规划，做大做强汽车零部件、机械电子制造、建材原材料、医药化工、农副产品深加工等主导产业。进一步发展光电、卫生洁具等产业集群。培育和发展新能源、节能环保、新材料、新医药、信息产业等战略性新兴产业和高新技术产业。完善自主创新激励政策，推动企业技术中心等中心建设，加快电机、耐磨材料、不锈钢、特钢等质量检测检验中心建设。深化产学研合作，加强企业技术改造，促进传统产业优化升级。继续实施品牌战略，鼓励企业争创驰名商标、著名商标和名牌产品。继续确定部分对区域经济带动力强的骨干企业，协调帮扶，促进企业发展壮大。一如既往地支持中小企业发展。二是不断提升现代农业。继续实施农业产业化五年提升行动发展规划，着力推进规模化、组织化、标准化、品牌化建设。发展壮大家禽产业集群、竹产业集群、粮油加工产业集群。加快优质水稻示范园区建设，力争10个粮食产业园区主体工程竣工投产。加快茶叶、特色烟叶、蔬菜、水产等优质农产品标准化基地建设。积极推进油茶产业发展。大力发展农产品精深加工，开展“食品工业推进年”活动。加快土地承包经营权流转，发展适度规模经营。壮大龙头企业和农民专业合作社，完善产业化组织体系。培养一批能够带领群众致富的农村能人。积极建设现代农业园区。建立健全质量安全管理体系和基层为农服务体系。加强农产品市场营销，叫响一批特色明显的地方农产品品牌。三是加快发展现代服务业。高起点编制文化产业发展规划，积极打造宣城文化品牌。启动实施滕头村农业生态观光园、中国茶府、中国国际体育休闲中心、中国文房四宝产业园、宣酒文化园等项目。在市区开工建设五星级宾馆、大型购物中心，每个县市都要着手建设1—2个四星级以上宾馆、大型购物中心和文化产业项目。着手编制南漪湖旅游开发规划。积极发展休闲度假游、历史文化游、名人故里游、乡村生态游、红色教育游、特色工业游。继续开展城市形象和旅游产品的整体营销。主动对接上海世博会，营造“看世博、游宣城”的良好氛围。加强与周边景区的合作，共建皖南国际旅游文化示范区。积极发展电子商务和现代物流业。启动双桥物流园区建设。加快宣城农副产品专业市场建设，培育1—2家省级大型农产品批发市场。启动会展经济。四是强力推进节能减排。积极发展低碳经济，推进高碳产业低碳化改造和低碳技术应用。加快发展循环经济，实施一批节能和资源综合利用工程。进一步加大落后产能淘汰力度，整治关闭小钢铁、小煤矿、小水泥，逐步淘汰粘土砖瓦窑。以列入全国千家、省百家节能行动的9户企业为突破口，突出抓好重点耗能企业节能降耗工作。认真抓好公共机构节能工作。严格环境评价制度，强化建设项目新增排放容量管理，加强对重点减排项目的监管，严禁新上对生态环境破坏和污染的项目。必须把加快污水处理配套管网建设作为一件十分重要而紧迫的事来抓，确保污水处理厂正常运行。

　　第三，坚定不移抓基础设施，尽力增加承接容量。以高速铁路、高速公路、水运航道建设为重点，加快完善综合交通网络，构建与长三角地区的快速连接通道，增强产业和资本的可进入性。积极协助做好合福铁路、黄杭铁路客运专线建设、皖赣铁路扩能及电气化改造工作，全面启动合杭铁路客运专线项目前期工作。积极推进宣城至宁国、宁国至绩溪、绩溪至黄山和芜雁高速公路建设，争取宣南铜高速早日复工。实施广祠高速路面改造、宣广高速宣城东西出入口改建。实施宣城至狸桥、宣城至泾县、宣城至古泉公路快速通道建设和梅岭隧道以及连接线工程。加快推进芜申运河宣城段建设。力争宁国至千秋关高速、溧阳至广德高速、水阳江航道整治和绩溪至长安公路改建工程开工。确保广德至双桥、蔡家桥至谭家桥、祠山岗至界牌和华龙公路竣工。调整市区客运站场规划布局，新建汽车客运东站和西站。

　　继续按照“五个起来”的要求，强力推进城市建设，完善城市功能，确保市本级完成城市基础设施建设投资25亿元以上。进一步加强城乡规划工作，更好地发挥规划的调控作用。续建滨湖路、环城大道、向阳大道、水阳江大道等10条城市道路，实施宝城路、薰化路、昭亭路、宛陵路等14条道路改建。继续抓好宛溪河、扬子鳄湖、敬亭山综合整治、梅溪公园建设和北门旧城改造等8个市政公用项目，新建3座下穿高速桥、3座上跨铁路立交桥等10个市政公用项目。继续推进城市防洪工程和天然气管网建设。启动宣州区政务新区建设的前期工作。开工建设市体育中心。推进宣城职业技术学院新校区二期工程、宣城中学新校区和市图书馆新馆建设，实施宣城二中扩建工程。加快数字化城管监管网络建设，继续开展市容市貌、市区交通秩序综合整治和禁违拆违工作，提升城市管理水平。加强住宅小区整治，完善物业管理机制，改善物业服务。加快城市环卫设施建设。

　　继续加强农村交通、水利、电力、通信等基础设施建设。完成公路“村村通”扫尾工作，加强农村公路管养。力争开工建设水阳江下游“开卡”工程。加快青弋江灌区续建配套与节水改造、大型泵站更新改造、病险水库除险加固等工程建设。实施500千伏广德变电站和5个110千伏输变电新建、扩建工程。把城镇化与新农村建设紧密结合起来，加大城镇建设力度，重点抓好县城和中心集镇建设。加快农民向城镇转移。推进新农村建设连点扩面工作，开展“美丽乡村”建设活动。

　　在加强基础设施建设的同时，进一步优化经济发展的软环境。完善考评机制和方法，深化民主考评活动，提升行政效能。继续推进行政审批权改革，强化政务服务中心功能，努力实现与长三角地区体制机制的对接。

　　第四，坚定不移抓深化改革，努力释放发展能量。把握政策机遇，找准结合点和切入点，不断深化重点领域改革。完善和落实各项优惠政策，破除体制机制障碍，促进非公经济进一步壮大。加快医药卫生体制改革，推进宣州区、郎溪县省级改革试点工作，稳步开展公立医院改革。加快文化体制改革，积极推进经营性文化单位转企改制和公益性文化事业单位内部改革，组建文化市场综合行政执法机构，加强对文化市场的有效监管。深化人口和计划生育综合改革，建立健全城市社区和流动人口综合管理服务工作机制，全面完成县乡计生服务站所标准化建设。深化农村各项改革。扩大县级经济社会管理权限。继续推进扩权强镇改革。扩大农村公益事业建设“一事一议”财政奖补试点范围。推进集体林权制度配套改革，加快林业服务平台建设。继续深化农村信用社改革，完成宣州、宁国、郎溪农村合作(商业)银行组建工作，推进村镇银行的组建。推进金融产品创新。探索建立信贷担保机制，扩大农村有效担保物范围。引导有条件的农民专业合作社在产品合作基础上，开展资金信用合作。继续推进政策性农业保险工作。全面完成县(市、区)政府和乡镇机构改革。积极推进人事制度改革，全面实施事业单位岗位设置管理。完成事业单位绩效工资改革工作。加强人才的培养和使用，建立健全创新人才引进和流动机制，吸引更多的人才来宣发展。积极培育人才市场和劳务市场。

　　第五，坚定不移抓市区一体化，合力做大本级体量。进一步加大市区联动、共同发展的力度，通过抓开发区、抓具体项目、抓工作调度、抓实战能力提升等等，持续推进市本级发展。以增量提速、扩容提质为主线，加快市开发区建设。“全面主攻西扩新区、提升完善宣南片、加快推进莲塘片”，努力做到道路连通、功能配套。启动实施“聚焦10户重点企业、培育20户优质企业、促进100户小企业进入规模企业”三年计划。力争今年工业总产值、规模工业增加值、财政收入增长30%以上；固定资产投资、工业投资增长50%以上；利用内外资、新落地企业数增长70%以上。合理规划市、区经济开发区的产业布局，明确功能定位，整合市、区土地、人才、资金和信息等优势，推动招商引资、项目建设统一规划、错位发展，实现市、区之间优势互补、项目互动。支持宣州开发区扩大建成规模，提升建设品质。继续下大力气积极争取国投电厂二期工程。完善市、区两级管理体制，建立一体化发展的协调机制。

　　第六，坚定不移抓民生改善，更加凝聚社会力量。认真落实中央和省着力保障和改善民生的要求，从群众最盼望、受益最快最多、感受最直观的地方着手，推进民生工程，发展社会事业。精心组织实施省定33项民生工程，让城乡居民共享更多的改革发展成果，进一步增强群众的安全感、幸福感和自豪感。以创业促就业，健全创业服务和公共就业服务体系，统筹做好农民工、高校毕业生、下岗失业人员就业和退役军人安置工作，确保新增城镇就业2万人。加强创业园区建设。广泛开展就业和创业培训。继续完善社会保障制度。开展新型农村社会养老保险试点。确保被征地农民养老金按时足额发放。继续做好城乡低保和医疗救助提标扩面工作。全面落实强农支农惠农政策，确保惠农补贴资金及时足额到位。加强扶贫开发工作。继续推进义务教育均衡发展，普及高中阶段教育，大力发展职业教育，规范学前教育。启动筹建本科院校的争取工作。开展新一轮中小学布局调整，继续实施“高中进城”计划。做好旌德双和职业技术学院建设前期工作。开展“中小学办学行为规范年”活动。切实加强中小学生就学安全管理。广泛开展全民健身活动，实施城乡体育健身工程。积极备战省十二运会。完善重大疾病防控体系。加强社区和农村卫生服务体系建设，继续实施乡镇卫生院改扩建工程。扩大新农合受益面，提高补助标准。切实加强公民思想道德建设和精神文明创建工作。加快乡镇综合文化站和农家书屋建设。完成广播电视“村村通”建设任务。继续实施数字电视整体转换。继续发展老龄事业、残疾人事业和慈善福利事业，切实保障妇女和未成年人合法权益。

　　继续加强“法治宣城”建设，争创全国“五五”普法先进城市。认真落实社会治安综合治理各项措施，加大财政投入，推进科技强警，严厉打击黑恶势力和各类违法犯罪行为，打造“平安宣城”。引导群众依法表达合理诉求，大力开展矛盾纠纷排查调处工作，妥善解决群众反映强烈的突出问题，维护社会和谐稳定。加快“生态宣城”建设，开展国家生态市创建工作，争创国家森林城市和环保模范城市，继续推进国家和省级环境优美乡镇、生态村创建工作。加强村镇环境综合整治，开展村镇生活污水和生活垃圾处理设施建设试点工作，强化对农村畜禽养殖污染整治。

　　构建保障有力、保护严格的土地利用新机制，严防违法占用耕地面积超过15%的问责“红线”。加快推进农村土地确权登记发证工作。大力推进农村土地整治、增减挂钩和土地置换工作。积极防治地质灾害。认真开展第六次全国人口普查，完成第二次全国经济普查工作。加强安全生产基层基础工作，加大隐患排查治理和重大危险源防控力度，严防较大以上事故发生。强化食品药品质量安全监管。支持工会、共青团、妇联等人民团体参与社会管理和公共服务。加强社区管理，提高社区建设和服务水平。支持驻宣部队建设。加强国防教育、国防动员和民兵预备役工作，继续开展“双拥”共建活动。加强应急平台建设，完善防震减灾、应急管理和保障机制，提高应对处置能力。认真做好民族宗教、侨务外事、对台、档案、地方志等工作。

　　三、推进政府自身建设

　　繁重的发展任务、激烈的竞争态势，对政府工作提出了新的更高的要求。我们必须进一步加强政府自身建设，思想再解放、机制再创新，强化执行力、保持快节奏，切实增强政府工作的实效性，不断提高人民群众对政府工作的满意度。

　　(一)进一步推进政府工作项目化。实践证明，任何一个地方的发展，都是由一个又一个项目支撑起来的，大项目带来大发展，小项目只能小发展，没项目就没发展。可以说，项目工作贯穿于经济和社会发展的全过程，体现在政府工作的多方位。比如，发展工业就要抓工业项目，发展农业就要抓农业项目，发展旅游就要抓旅游项目，发展文化产业就要抓文化产业项目，交通状况要改善就要抓交通项目，城市面貌要改变就要抓城建项目，等等。因此，各级各部门都要切实增强项目意识，把项目工作放在更加突出的位置来抓，不断提升项目谋划、项目争取、项目推进、项目实施的能力和水平，“让干部用项目说话，让项目为干部说话”。

　　(二)进一步推进政府工作具体化。众所周知，政府工作必须了解具体情况，研究具体招法，处理具体事务，解决具体问题。因此，只有具体才能深入，只有具体才能主动，只有具体才能见效。如果不把握抓具体的要义，工作不细致、不扎实、不艰苦，谈问题似是而非，讲道理肤浅表面，抓工作大而化之；如果只讲书上怎么说、不谈自己怎么看，只有原则怎么样、没有具体怎么办；如果误认为会议开了、任务就落实了，文件下了、事就办了，话说过了、就做到了，这样的话，政府就难以有作为，事业就难以有发展。当前，我们面临的大事多、难事多。事多，应该成为我们拼命工作的理由，而不能作为顺其自然的托辞；事难，应该成为我们兢兢业业的根据，而不能作为敷衍了事的借口。我们必须倡导具体抓、抓具体，抓住不放、一抓到底的作风。

　　(三)进一步推进政府工作责任化。经验表明，一个人的进步需要有责任心，一个地方的发展同样需要有责任心。责任心虽然无形无状、难触难摸，但是力量巨大、影响深远。有责任心的人，受人尊重、招人欣赏、让人放心。政府所有人员都要坚持守土有责，把解放思想、按规矩办事和敢于负责、勇于担当有机结合起来，主动作为、善于作为。如果只讲解放思想，而不敢承担责任，就是“口头派”，是不受欢迎的人；如果只讲按规矩办事，而不敢创新，就是“机器人”，是不称职的人；如果只讲大胆开拓，而不能坚守原则，就是“冒失者”，是不可靠的人。该是哪个地方负责的，就由哪个地方负责；该是哪个部门负责的，就由哪个部门负责；该是哪个层次负责的，就由哪个层次负责。我们坚决反对上一级向下一级推卸责任、揽功诿过，也坚决防止下一级明哲保身，甚至有意无意让上一级承担责任，我们也要坚决克服总是要求别人解放思想，自己却想方设法解脱责任的倾向。我们将进一步严格责任制和责任追究制，加大行政问责力度，做到“抓住不落实的事，追究不落实的人”。我们相信，只要人人更加负责，宣城的前景一定会更加美好。

　　(四)进一步推进政府工作全员化。大家都有深切体会，一部机器如果要正常运行，就需要每一个零部件都发挥作用，哪怕一个小零件出了问题，都可能发生“停车断档”。做好政府工作如同一部机器的运行一样，离不开每一位政府人员的爱岗敬业、恪尽职守。正如毛泽东同志曾经指出：“世界是我们的，做事要大家来。”宣城的跨越崛起，是一项需要全市上下倾心奋斗的事业，人人都应该是“责任人”，而不是“局外人”；个个都应该是“当事者”，而不是“旁观者”。政府人员特别是领导干部，更应该自觉地接受人大的法律监督、政协的民主监督、各民主党派和工商联的监督以及群众监督和新闻舆论监督，脚踏实地、心无旁骛、认真负责地把分内事做好，做到“我的岗位我负责，我的工作你放心”，人人有激情，天天在状态，满腔热忱为群众解难，真心实意为基层办事。

　　各位代表，各位同志，前进的道路充满挑战，肩负的责任重如泰山。我们坚信，任何困难都改变不了我们富民强市的强烈愿望，任何挑战都阻挡不住我们跨越崛起的坚定步伐。让我们在中共宣城市委的坚强领导下，把人民群众的期望化为阔步前进的动力，把加快发展的使命变为脚踏实地的行动，为实现宣城的科学发展、和谐发展、跨越发展而努力奋斗！
